# Differential Regulation of the Ribosomal Association of mRNA Transcripts in an *Arabidopsis* Mutant Defective in Jasmonate-Dependent Wound Response

**DOI:** 10.3389/fpls.2021.637959

**Published:** 2021-03-11

**Authors:** Athen Kimberlin, Rebekah E. Holtsclaw, Abraham J. Koo

**Affiliations:** ^1^Department of Biochemistry, University of Missouri, Columbia, MO, United States; ^2^Interdisciplinary Plant Group, University of Missouri, Columbia, MO, United States

**Keywords:** translatome, wound, JA-Ile, jasmonate, CYP94B, ribosomal profiling

## Abstract

Jasmonoyl-L-isoleucine (JA-Ile) is a powerful oxylipin responsible for the genome-wide transcriptional reprogramming in plants that results in major physiological shifts from growth to defense. The double T-DNA insertion *Arabidopsis* mutant, *cyp94b1cyp94b3* (*b1b3*), defective in cytochrome p450s, CYP94B1 and CYP94B3, which are responsible for oxidizing JA-Ile, accumulates several fold higher levels of JA-Ile yet displays dampened JA-Ile–dependent wound responses—the opposite of what is expected. Transcriptomic and proteomic analyses showed that while the transcriptional response to wounding was largely unchanged in *b1b3* compared to wild type (WT), many proteins were found to be significantly reduced in the mutant, which was verified by immunoblot analyses of marker proteins. To understand this protein phenotype and their hypothesized contribution to the *b1b3* phenotypes, wounded rosette leaf samples from both WT and *b1b3* were subject to a translating ribosome affinity purification RNA sequencing analysis. More than 1,600 genes whose transcripts do not change in abundance by wounding changed their association with the ribosomes after wounding in WT leaves. Consistent with previous observations, the total pool of mRNA transcripts was similar between WT and *b1b3*; however, the ribosome-associated pool of transcripts was changed significantly. Most notably, fewer transcripts were associated with the ribosome pool in *b1b3* than in WT, potentially explaining the reduction of many proteins in the mutant. Among those genes with fewer ribosome-associated transcripts in *b1b3* were genes relating to stress response, specialized metabolism, protein metabolism, ribosomal subunits, and transcription factors, consistent with the biochemical phenotypes of the mutant. These results show previously unrecognized regulations at the translational level that are affected by misregulation of JA homeostasis during the wound response in plants.

## Introduction

Much progress has been made in understanding the genome-wide transcriptional reprogramming after wounding. It has been estimated that several hundred to a few thousand genes change their expression upon wounding. and a majority of those genes are attributed to the jasmonate (JA) pathway (Reymond et al., 2000; Pauwels et al., 2009; Bhosale et al., 2013; Attaran et al., 2014; Hickman et al., 2017; Ikeuchi et al., 2017; Zander et al., 2020; Aerts et al., 2021). Among the changing genes are genes relating to the defense traits of plants against insects and pathogens such as those involved in production of defense compounds (Howe and Jander, 2008; Wasternack and Hause, 2013) and genes relating to growth inhibition (Zhang and Turner, 2008; Wu and Baldwin, 2010; Huot et al., 2014). The final outcome of these coordinated changes, which is commonly referred to as the defense versus growth trade-off, is expected to be complex and may come either as a passive result of the sum of the changes or an actively regulated process (e.g., cell cycle progression interference or hardwired transcriptional network) or both (Noir et al., 2013; Campos et al., 2016; Havko et al., 2020).

At the center of this transcriptional reprogramming is the biosynthesis of jasmonoyl-L-Ile (JA-Ile), a bioactive metabolite form of JA, which can activate transcriptional changes through physical binding to a nuclear-located hormone receptor co-complex consisting of CORONATINE INSENSITIVE1 (COI1) and JASMONATE ZIM-domain (JAZ) proteins (Chini et al., 2007; Thines et al., 2007; Yan et al., 2007; Fonseca et al., 2009; Sheard et al., 2010; Howe et al., 2018). The physical binding of JA-Ile with COI1-JAZ ultimately results in activation of transcription factors that in turn promotes transcription of hundreds to thousands of JA-responsive genes. As part of our investigation to understand how this pathway is inactivated, two genes belonging to the CYP94 clade of *Arabidopsis* cytochrome P450 enzymes, CYP94B1 and CYP94B3, were identified (Kitaoka et al., 2011; Koo et al., 2011, 2014; Heitz et al., 2012). These enzymes act as JA-Ile-12-hydroxylases in the so-called ω-oxidation pathway that oxidizes JA-Ile in a sequential manner to eventually catabolize the hormone (Koo and Howe, 2012; Koo, 2018). In support of the role of these enzymes in JA-Ile turnover, *Arabidopsis* plants overexpressing either enzyme display reduced JA-Ile content and associated JA-deficient phenotypes (Koo et al., 2011, 2014). Recently, in maize, it was discovered that the genetic lesion responsible for the feminized tassels of the classical *Tasselseed5* mutant results from overexpression of *ZmCYP94B1* that depletes the bioactive pool of JA-Ile that is required to abort silks in the tassel (Lunde et al., 2019), consistent with the role of CYP94s in JA-Ile turnover.

Conversely to the overexpression, *Arabidopsis* double homozygous T-DNA insertion mutant *cyp94b1cyp94b3* (*b1b3*) overaccumulates JA-Ile due to the blockage of its turnover (Poudel et al., 2016). However, in contrast to the straightforward biochemical phenotypes of the CYP94B overexpressing mutants, puzzling results were obtained while analyzing the *b1b3* mutant (Poudel et al., 2016). Despite the increased level of bioactive JA-Ile [at the expense of 12-hydroxy-JA-Ile (12OH-JA-Ile), the product of CYP94Bs catalysis] in this mutant, the plants displayed a range of phenotypes that are more typical of plants lacking JA-Ile. For example, *b1b3* plants were more susceptible to insect attack, were more resistant to the growth inhibitory effects of wounding, accumulated fewer trichomes, and experienced a global reduction in specialized metabolites when compared to wild type (WT) (Poudel et al., 2016). Exogenous JA application and following transcriptomic analysis confirmed that these phenotypes were not due to *b1b3* being insensitive to JA and that the perception of JA and JA-regulated transcription was happening normally in the mutant. Subsequent studies have revealed bioactivity of 12OH-JA-Ile similar to that of JA-Ile (Jimenez-Aleman et al., 2019; Poudel et al., 2019). Exogenous 12OH-JA-Ile induced a large number of JA-Ile–inducible genes, promoted anthocyanin and other specialized metabolite accumulation, increased the number of trichome cells, inhibited root growth, and reduced insect larvae growth, and its signaling was blocked by mutation in COI1 (Jimenez-Aleman et al., 2019; Poudel et al., 2019). Based on these findings, it was suggested that the lack of 12OH-JA-Ile in *b1b3* may contribute to its attenuated wound response (Poudel et al., 2019). This explanation was supported by genetic analysis of several pathway-engineered plants that either mimicked or offset the JA profile of *b1b3* (Poudel et al., 2019). Even though the surprisingly strong impact of altered 12OH-JA-Ile levels *in planta* was discovered in that study, they still did not completely resolve the problem of why *b1b3*, containing a more than threefold higher level of JA-Ile, shows a weakened wound phenotype (Poudel et al., 2016).

These collective observations and the additional discovery of proteome changes described in this article have led us to an unbiased genome-scale studies in the *b1b3* mutant. In particular, we have applied a technique known as translating ribosome affinity purification RNA sequencing (TRAP-Seq) (Zanetti et al., 2005) to study JA and wound signaling in plants. The collective multiomics results provide potential explanations for phenotypic changes in *b1b3* and also reveal previously unrecognized regulations at the translational level in response to wounding in *Arabidopsis*.

## Materials and Methods

### Plant Materials, Growth Conditions, and Wounding Treatment

All plants were grown under long day conditions (16-h light, 100–120 μE m^–2^ s^–1^) in growth chambers kept at 22°C. *Arabidopsis thaliana* ecotype Columbia-0 (Col-0) was used as the WT, and the double T-DNA insertion mutant *cyp94b1-1cyp94b3-1* (*b1b3*) was described earlier (Koo et al., 2014). Plants used for TRAP-Seq experiments harbor a transgene consisting of the 6X histidine-FLAG-epitope tagged ribosomal protein (RP) L18 (HF-RPL18) gene under a cauliflower mosaic virus (CaMV) 35S promoter (35S:HF-RPL18). The WT seeds harboring 35S:HF-RPL18 were a gift from Dr. David Mendoza from the University of Missouri, who received them from previously published work (Mustroph et al., 2009). The binary vector (pGreen2 35S:HF-RPL18) used to transform *b1b3* was also a gift from the Mendoza laboratory. Plant transformation was done according to the floral dip method (Clough and Bent, 1998). Seeds harvested from the resulting plants (T1) were screened for resistance to hygromycin (50 μg mL^–1^) and expression of RPL18.

Wounding treatment was executed by firmly pressing a pair of serrated-tip hemostats across the leaf midvein three times, with two leaves per plant being wounded in general. For anthocyanin measurements and immunoblot for systemic induction of marker proteins, plants were wounded according to the wound-induced growth inhibition (WIGI) wounding scheme described previously (Poudel et al., 2016). Data generated from two biological replicates for proteomics and three biological replicates for transcriptomics and TRAP-seq were used in this study. All tissues were collected at their respective time points and immediately frozen in liquid nitrogen and stored in −80°C until use. The anthocyanin measurement method was performed as described (Poudel et al., 2016).

### Protein Extraction, Immunoblots, and Proteomics

Rosette leaves from untreated (control, unwounded) or wounded (8 h) 3- to 4-week-old WT and *b1b3* plants were used as tissue to extract proteins. Frozen tissue was ground to a powder in a prechilled mortar and pestle, and protein was extracted with 50 mM sodium phosphate buffer, pH 7.0, containing 10% glycerol, 50 mM NaCl, and protease inhibitor tablets (Pierce/Thermo Fisher Scientific). Samples were briefly spun down to remove debris. Proteins were quantified by the bicinchoninic acid method (Pierce/Thermo Fisher Scientific).

Equal amounts (20 μg) of protein were loaded and separated by sodium dodecyl sulfate–polyacrylamide gel electrophoresis (SDS-PAGE). Proteins were transferred to polyvinylidene fluoride membrane overnight at 4°C at constant 40 mA. Anti-LOX2 (Agrisera, Sweden) antibody was used at a 1:15,000 dilution, whereas anti-JAR1 (Cocalico Biologicals Inc., PA) and Anti-AOC [gift from Dr. Bettina Hause, Leibniz Institute of Plant Biochemistry, Halle (Saale), Germany] were used at a 1:3,000 dilution. Secondary antibody was horseradish peroxidase–conjugated ECL anti–rabbit immunoglobulin G from donkey (GE Healthcare) and was used at a 1:15,000 dilution. Blots were visualized using SuperSignal West Pico Chemiluminescent Substrate (Pierce/Thermo Fisher Scientific) and x-ray films (Midwest Scientific, MO).

Approximately 120 μg of protein was used per sample for proteomics analysis. The samples were acetone precipitated, and protein pellets were resuspended in 100 mM ammonium bicarbonate, 6 M urea, and 2 M thiourea. Samples were reduced in 200 mM DTT for 1 h and alkylated in 200 mM iodoacetamide for 1 h. Unreacted iodoacetamide was consumed by addition of excess DTT. Water was then added to dilute urea in samples to ∼0.6 M. Trypsin (Promega, WI, United States) was then added at a ratio of ∼1:50 (protease:substrate), and digestion was carried out at 37°C overnight. The reaction was stopped by the addition of formic acid (1% vol/vol). Sample peptides were then purified and concentrated by C18 tip (Pierce/Thermo Fisher Scientific). Samples were lyophilized and resuspended in 5:1% acetonitrile:formic acid, and data acquired on a Bruker timsTOF pro at the University of Missouri Gehrke Proteomics Center. Approximately 0.8 μg of suspended peptide was separated on Bruker nanoElute fifteen C18 ReproSil AQ column (150 mm × 75 μm, 1.9 μm) (packing material from Dr. Maisch GmbH, Ammerbuch-Entringen, Germany) with a step gradient of acetonitrile at 400 nL min^–1^ flow rate. The Bruker nanoElute system is connected to a timsTOF pro mass spectrometer. Initial LC gradient conditions were as follows: 3% B (A: 0.1% formic acid in water, B: 99.9% acetonitrile, 0.1% formic acid), followed by 51-min ramp to 30% B, 30–50% B over 7.5 min, gradient of 50% B to 80% B over 5.5 min, held at 80% B for 6 min with total run time of 70 min. MS data were collected over an *m/z* range of 100 to 1,700. During MS/MS data collection, each TIMS cycle included 1 MS an average of 10 PASEF MS/MS scans. The acquired data were submitted to the PEAKS X+ search engine for protein identifications against TAIR10 protein database. Data search parameters included trypsin as enzyme, two missed cleavages allowed; carbamidomethyl cysteine as a fixed modification; oxidized methionine and deamidation of asparagine and glutamine as variable modification; 50 ppm mass tolerance on precursor ions; and 0.5 Da on fragment ions. The PEAKS X+ search estimates false discovery rate (FDR) using a “decoy fusion” approach (Zhang et al., 2012). Data were filtered for peptide-spectrum match (PSM)–FDR of < 0.1%. PSM-FDR is the total number of decoy database assignments to spectra relative to the total number of target database assignments to spectra represented as a percentage. Entry with an average spectral count of two or more in at least one of the three treatment replicates was included, and fold change (FC) of 2 was applied as cutoff.

### Translating RNA Affinity Purification

The method of isolating the FLAG-tagged ribosomes was described previously (Zanetti et al., 2005; Castro-Guerrero et al., 2016). Briefly, frozen tissue samples were ground using a mortar and pestle yielding approximately 10 to 15 g of powdered tissue per sample. Ice-cold ribosome extraction buffer (200 mM Tris-HCl pH 9.0, 200 mM KCl, 25 mM EGTA, 36 mM MgCl_2_, 1 mM DTT, 50 μg mL^–1^ cycloheximide, 50 μg mL^–1^ chloramphenicol, 1 mM phenylmethylsulfonyl fluoride, 1% igepal CA-630, 1% Brij 35, 1% triton X-100, 1% tween 20, 1% tridecyl ether, 1% sodium deoxycholate, 0.5 mg mL^–1^ heparin) was added at a 2:1 ratio (buffer:tissue). The samples were gently rocked at 4°C for 30 min. The samples were then centrifuged at 16,000 × *g* for 15 min at 4°C. The supernatant was gently filtered through miracloth and collected in a separate tube. To the supernatant, 300 μL of EZ view Red Anti-FLAG M2 Affinity Gel (Sigma) was added and incubated with gentle rocking for 2 h at 4°C. Samples were briefly centrifuged to recover beads and were washed four times with 10 mL of wash buffer (200 mM Tris-HCl pH 9.0, 200 mM KCl, 25 mM EGTA, 36 mM MgCl_2_, 1 mM DTT, 50 μg mL^–1^ cycloheximide, 50 μg mL^–1^ chloramphenicol). Beads were collected by centrifugation, and RNA was extracted immediately from them using the Direct-zol RNA MiniPrep Plus Kit (Zymo Research, CA, United States). RNA samples were normalized to ∼100 ng mL^–1^, and approximately 3 μg of RNA was sent to Novogene Corporation Inc (Sacramento, CA) for RNA sequencing (RNA-Seq) analysis.

### Total RNA Isolation and RNA-Seq

Approximately 15 g of rosette leaf tissue from either wounded or untreated (unwounded) plants was ground up with prechilled mortar and pestle while frozen in liquid nitrogen. A 50-mg aliquot of the ground tissue was used for total RNA extraction, and the remaining tissue was used for the TRAP experiment. The total RNA was extracted using the Direct-zol RNA MiniPrep Plus Kit (Zymo Research, CA, United States) and was treated with TURBO DNA-free kit (Thermo Fisher Scientific, MA, United States). Both total and TRAP RNA was sent for sequencing by Novogene (Sacramento, CA), which describes using the following procedures. Briefly, after the standard RNA quality control assessments, mRNA was enriched using oligo(dT) beads after which the cDNA library was constructed. Library concentration was first quantified using a Qubit 2.0 fluorometer (Life Technologies) and then diluted to 1 ng μL^–1^ before checking insert size on an Agilent 2100 and quantifying to greater accuracy by quantitative polymerase chain reaction (qPCR). Libraries were fed into respective Illumina sequencers according to activity and expected data volume. Raw reads were filtered to remove reads containing adapters or reads of low quality, so that downstream analyses were based on clean reads. The sequences were mapped to the reference genome using Tophat2 (Kim et al., 2013) with the mismatch parameter set to 2 and all others set to default. Read counts were normalized and used for differential expression using DEseq (Anders and Huber, 2010). Negative binomial distribution after normalization by DEseq (adjusted *P* value) of less than 0.05 and log_2_ FC of 1 (twofold) was used to determine differential expression.

### qPCR

One microgram of RNA from each biological replicate from both the total and TRAP samples was reverse transcribed using oligo(dT)_20_ primers and Moloney murine leukemia virus reverse transcriptase (Promega, WI, United States) and was used as the template for semiquantitative reverse transcription (qRT)–PCR reaction using SYBR^TM^ Green PCR Master Mix (Thermo Fisher Scientific, MA, United States) in a CFX96 Touch^TM^ real-time PCR detection system (Bio-Rad, CA, United States). ACTIN8 (AT1G49240) was used as an internal reference gene, and the relative transcript abundance was expressed as FC relative to the mock treatment. Primers (5′-3′) used for qRT-PCR were JAZ7qPCRF: CGACTTGGAACTTCGCCTTCTTA, JAZ7 qPCRR: ACATCTCTACTCGCTAGCGATAG; JAR1qPCRF: CA TCGATGTCTCGACAGATCCAGGA, JAR1qPCRR: GCTCC AAGGCTCCAATAGTCTTGC. Primers for *OPR3* and *ACTIN8* have been described earlier (Poudel et al., 2016).

### Data Mining

For data analysis and visualization, the following web-based programs and public databases were used. For area proportional Venn diagrams, BioVenn was used^1^ (Hulsen et al., 2008). For hierarchical clustering and heatmapping exercises, the online tool, Morpheus^2^, from the Broad Institute, Cambridge, MA, United States, was used. For cluster analysis, Euclidean distance with complete linkage was used. Correlation coefficient chart was also generated using Morpheus tool. The program MapMan^3^ (Thimm et al., 2004) was utilized for pathway mapping and visualization using the most up-to-date TAIR10 genome. For further pathway analysis, Kyoto Encyclopedia of Genes and Genomes (KEGG) Pathway Analysis tools were used^4^. Gene Ontology (GO) terms were assigned by the protein analysis through evolutionary relationships classification system, Protein ANalysis THrough Evolutionary Relationships (PANTHER) version 14^5^ (Mi et al., 2018). All GO term categories were found using the GO aspect, GO biological process, except for the categories for up-regulated WT_U_TRAP/WT_U (Figure 4), where the GO aspect, PANTHER GO-Slim Biological process was used. All GO term datasets were calculated using the Fisher exact test and Bonferroni correction for multiple testing.

## Results

### There Is a Global Reduction in the Proteome of Wounded b1b3 Leaves Compared to WT

**FIGURE 1:**
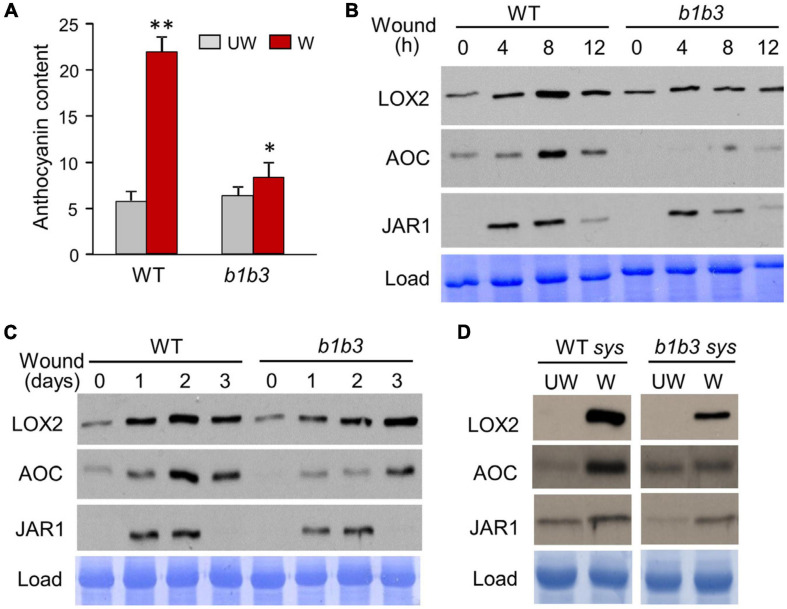
Wound-inducible proteins accumulate to a lesser extent in *b1b3*. **(A)** Anthocyanin levels in wounded (W) and unwounded (UW) WT and *b1b3* plants. Plants were wounded multiple times following the WIGI (wound-induced growth inhibition) wounding scheme described in the section “Materials and Methods.” Bar graphs represent means ± SD (*n* = 5). Asterisks denote statistical significance by Student *t* test compared to UW WT: ^∗^*P* < 0.05, ^∗∗^*P* < 0.01. **(B)** Immunoblots showing a 12-h wounding time course of LOX2, AOC, and JAR1 in WT and *b1b3*. Fully expanded rosette leaves of 25-day-old plants were crushed two times across the midrib per leaf with a hemostat. **(C)** A 3-day wound time course showing lower protein accumulation for *b1b3*. Two leaves per plant were wounded once every day for 3 days and samples collected 12 h after wounding each day. **(D)** Protein accumulation in the undamaged systemic leaves of unwounded (UW) and wounded (W) WT and *b1b3* plants. Plants were wounded according to the WIGI wounding scheme as in **(A)**. Remaining undamaged leaves were harvested 1 day after final wounding and used as systemic samples.

It has previously been observed through multiple biochemical and physiological studies that much of the downstream JA-dependent wound responses including anthocyanin accumulation (Figure 1A) are down-regulated in the *cyp94b1cyp94b3* (*b1b3*) mutant when compared to WT despite containing threefold to fourfold higher levels of JA-Ile in the mutant (Koo et al., 2014; Poudel et al., 2016). Gene expression studies showed either similar or increased levels of mRNA transcripts for several JA-responsive marker genes in the wounded leaves of *b1b3* (Poudel et al., 2016), making it difficult to explain the phenotype by differential gene expression. In an attempt to close the gap in our understanding between transcription and downstream responses in this mutant, protein immunoblots were carried out (Figures 1B–D). Total proteins extracted from wounded leaf samples collected 4, 8, and 12 h after wounding along with unwounded (0 h) leaves from WT and *b1b3* were separated on an SDS-PAGE gel and probed with antibodies against three wound response marker proteins in the JA pathway, LOX2, AOC, and JAR1. All three proteins detected were induced by wounding in 4 h, peaking around 8 h (Figure 1B). Surprisingly, all three marker proteins were recognizably reduced in *b1b3* compared to WT. A variation of wound time course was carried out over 3 days (Figure 1C). In this case, the same set of leaves (two leaves per plant, leaf numbers 3 and 4) was wounded once every day for 3 days, and leaves were collected 12 h after wounding each day. This type of wounding had been used for WIGI assays before (Poudel et al., 2016), which resulted in differential growth suppression between the two genotypes. Immunoblot signals for LOX2 and AOC intensified until days 2 and 3 in the WT and *b1b3*, respectively (Figure 1C). Importantly, signals for all three markers were either weaker or delayed in *b1b3* compared to WT, similarly, to the short time course (Figure 1B). Marker protein changes in the systemic leaves of the wounded plant were also assessed (Figure 1D). Most of the differential response to wounding in *b1b3* was attributed to systemic wound signaling (Poudel et al., 2016). Clear induction of proteins in the systemic leaves was detected for LOX2 and AOC. Systemic induction of JAR1 was less obvious. However, both LOX2 and AOC induction was weaker in *b1b3* than in WT (Figure 1D).

**FIGURE 2:**
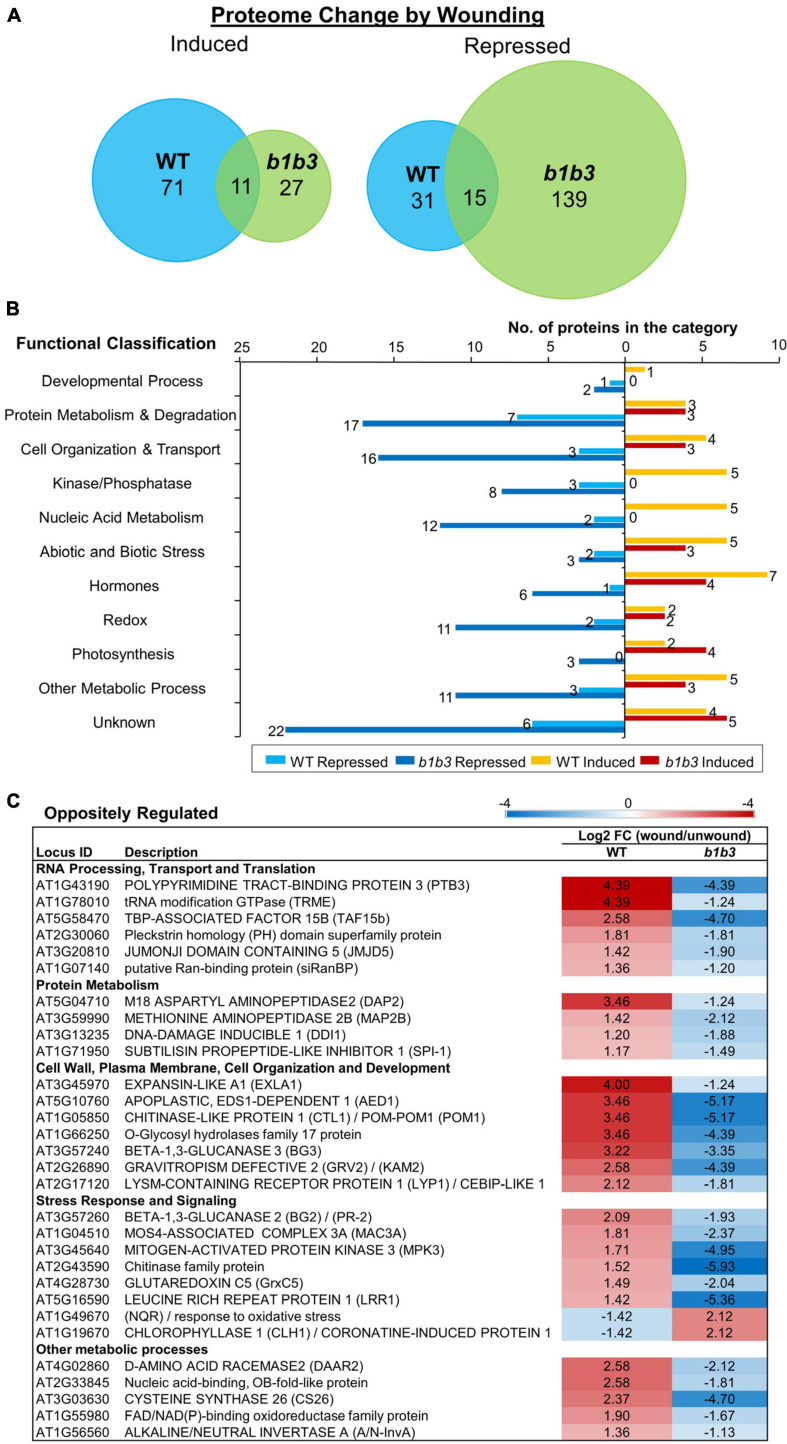
Protein changes by wounding in WT and *b1b3* leaves. **(A)** Area-proportional Venn diagrams showing number of proteins induced or repressed twofold or more by wounding compared to unwounded leaves of WT and *b1b3.* **(B)** Functional classification of proteins differentially regulated by wounding [fold change (FC) ≥ 2]. GO assignment was based on TAIR. **(C)** List of oppositely regulated proteins in WT and *b1b3* grouped into functional categories. The values are −1 ≥ log_2_ FC (wound/unwound) ≥ 1 and displayed as a heatmap. Average spectral counts from LC-MS/MS analysis of two biological replicates were used to calculate the FC.

The protein markers used so far are limited to the JA pathway, and so the next question was whether this phenomenon is limited to JA responsive proteins or is widespread across proteins outside the known JA regulatory network. To address this question, an unbiased proteomics experiment was carried out. Wounded leaf samples were collected 8 h after wounding based on earlier immunoblot results that showed the greatest discrepancy in protein amount between *b1b3* and WT (Figure 1B). Upon clearing of crude debris by medium speed centrifugation at 23,000×*g* for 45 min, all soluble fractions from both unwounded and wounded samples from WT and *b1b3* were analyzed by the nanoElute LC-timsTOF Pro mass spectrometer. A total of 1,956 proteins were identified with an average spectral count of two or more in at least one of the three treatment replicates with no other statistical constraints. Relatively low stringency criteria were used here in order to be more inclusive because our goal was to take a broader survey of the proteome rather than to identify specific proteins with a high level of confidence. The majority of proteins did not differ in spectral counts between WT and *b1b3*, but 204 (10%) in unwounded and 135 (6.9%) in wounded leaves were different by more than twofold between the two genotypes. These were mostly not the proteins encoded by those typically known as JA responsive genes (Reymond et al., 2000; Pauwels et al., 2008; Bhosale et al., 2013; Attaran et al., 2014; Poudel et al., 2019), indicating that the differentially regulated proteins in the mutant were not restricted to JA pathway. A comparable number (7%–10%) of proteins changed in abundance either up or down twofold or more in response to wounding in both genotypes (Figure 2A) (Supplementary Table 1). However, more proteins were notably repressed than induced by wounding in *b1b3* (Figure 2A). Compared to 82 induced and 46 repressed in WT, only 38 proteins were induced in wounded *b1b3*, whereas as many as 154 proteins were repressed in *b1b3* (Figure 2A). This is consistent with the downward trend of the marker protein levels observed by the immunoblots (Figure 1). Figure 2B displays the functional classification of 211 proteins induced or repressed by wounding. This clearly shows the predominant downward regulation in *b1b3* across all 11 functional categories. The top two known categories with the largest number of proteins were the “Protein Metabolism & Degradation” and “Cell Organization and Transport” classes. These may be related to the reduction in proteins and dampened growth responses to wounding in *b1b3* (Poudel et al., 2016).

Thirty proteins were even oppositely regulated between the two genotypes (Figure 2C). Interestingly, except for two that were repressed in WT, all the rest were repressed in *b1b3*, again reflecting the downward trend of protein abundance in wounded *b1b3*. These include several proteins involved in RNA processing and protein metabolism such as POLYPYRIMIDINE TRACT-BINDING PROTEIN 3 (Rühl et al., 2012) that showed the most extreme changes in abundance (log_2_FC (wound/unwound) of 4.39 in WT and -4.39 in *b1b3*). Additional genes that were oppositely regulated include those encoding pathogenesis-related proteins such as the BETA-1,3-GLUCANASES 2 (BG2), BG3 (Dong et al., 1991; Zavaliev et al., 2013), and APOPLASTIC EDS1-DEPENDENT 1 (Breitenbach et al., 2014). Together, these results show that there is a widespread reduction in proteome in the wounded leaves of *b1b3.*

### Many Transcripts Change Their Association With Ribosomes Upon Wounding in WT Leaves

**FIGURE 3:**
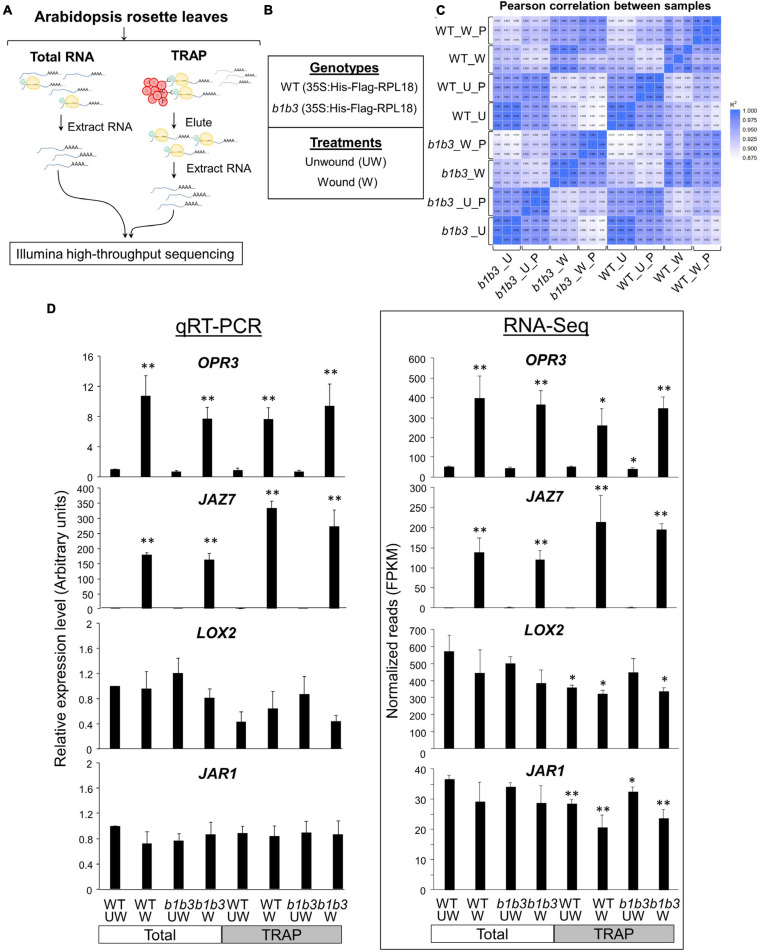
Overview of TRAP-Seq experiment and qPCR validation. **(A)** Schematics of total RNA-Seq and TRAP-Seq. Tissue samples were divided into two for total RNA extraction and TRAP. RPL18 of ribosomes (yellow) are epitope-tagged with 6X histidine (His)-FLAG (green), which is used to pull down by affinity to anti–FLAG-conjugated beads (red circles) to collect associated RNA. **(B)** Plant genotypes and treatments. **(C)** Pearson correlation between samples as an indicator of experiment reliability. The color gradient scale is shown where darkest blue indicates the *R*^2^ correlation value of 1 or perfect correlation. U, unwounded; W, wounded; P, polysomal. Each sample group labeled on the x- and y-axes consists of three sample replicates. Samples are ordered by experiment type. **(D)** Semiquantitative RT-PCR (qRT-PCR) (left) of *OPR3, JAZ7, LOX2*, and *JAR1* genes compared with RNA-Seq data (right). Transcript abundance is indicated by FPKM (fragments per kilobase of transcript sequence per million base pairs sequenced). *ACTIN8* was used as an internal reference gene, and the expression levels are displayed relative to the unwounded WT total RNA values. Data represent the mean + SE of three biological replicates. Asterisks denote statistical significance by Student *t* test compared to UW WT: ^∗^*P* < 0.05, ^∗∗^*P* < 0.01.

The distinctive proteomic features between WT and *b1b3* led us to wonder what might be the reason behind such difference. Protein abundance can be affected by both the synthesis of protein and the turnover of protein, so we hypothesized that the mRNA translation into protein may be differentially regulated in the two genotypes. Although less studied than the transcriptional responses, increasing evidence indicates changes occur at the level of translation in response to biotic as well as abiotic stresses (Branco-Price et al., 2005; Merchante et al., 2017; Merret et al., 2017; Sablok et al., 2017; Xu et al., 2017). Alterations in the translating pools of mRNA can have direct effects on protein abundance because increased association with the ribosomes increases the chance of them being translated. Although mRNA association with ribosomes does not always guarantee translation, it has been widely used as a proxy to gauge translational activity (Bailey-Serres et al., 2009; Reynoso et al., 2015; Mazzoni-Putman and Stepanova, 2018). In order to compare the relative levels of translation between WT and *b1b3*, a procedure called TRAP-Seq was employed (Reynoso et al., 2015). Briefly, RPL18, one of the subunits of the ribosomal complex, is tagged with an epitope tag (FLAG in our case) that can be used later to pull down the entire ribosomal complex bound with the mRNA from the total cell lysate using antibody-conjugated beads (Figure 3A). Total mRNA (bound and unbound to ribosome) and the ribosome-bound mRNA from wounded (20 min) and unwounded leaves of WT and *b1b3*, both harboring the FLAG-tagged RPL18, were subject to RNA-Seq analysis (Figure 3B). A 20-min wounding time point was used to capture early changes in ribosomal association that may result in later differences in protein levels. By 20 min, there is a significant increase in both JA hormone and early JA-responsive genes (Chung et al., 2008; Koo et al., 2009). For data analysis, we first studied wound-induced changes in WT followed by comparisons between the two genotypes. The total number of clean reads after filtering in each sample ranged from 74 to 113 million reads. For quality control measures, Pearson correlation coefficients for all biological replicates were calculated across all 24 sample replicates (Figure 3C). The darker the blue color is in the figure, the closer the R2 correlation value is to 1 or perfect correlation (squares aligned at the center diagonal line). All of the three biological replicates had an R2 correlation value greater than 0.96 (except one 0.875 and majority greater than 0.99) among themselves, showing low variation within sample replicates. RNA-Seq data were validated by qRT-PCR analyses of four marker genes, OXOPHYTODIENOATE-REDUCTASE 3 (OPR3), JASMONATE-ZIM-DOMAIN PROTEIN 7 (JAZ7), JASMONATE RESISTANT 1 (JAR1), and LIPOXYGENASE 2 (LOX2) (Figure 3D). Early genes, OPR3 and JAZ7, were strongly induced by wounding, whereas JAR1 and LOX2 transcripts were not increased upon wounding consistent with the delayed expression observed before (Reymond et al., 2000; Suza and Staswick, 2008; Koo et al., 2009; Body et al., 2019). In all cases, similar trends were seen between qRT-PCR and RNA-Seq data [expressed as fragments per kilobase of transcript per million mapped reads (FPKM)] for wounded and unwounded total and TRAP mRNA samples (Figure 3D).

**FIGURE 4:**
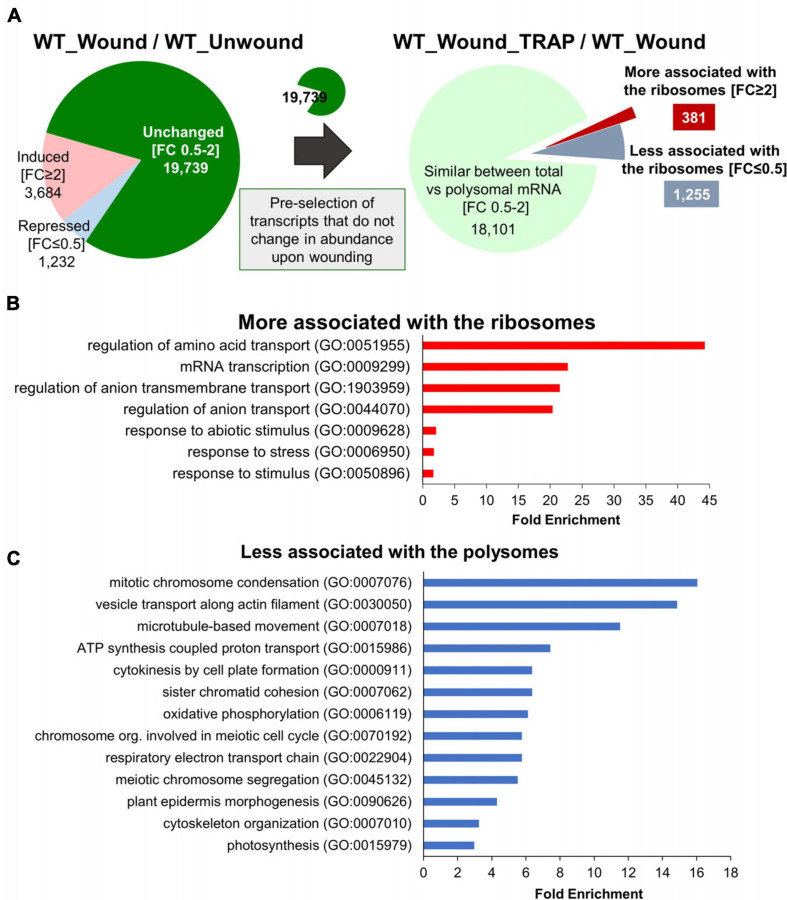
Genes with transcripts exclusively changing their ribosomal association after wounding without change in their net transcription. **(A)** Among 19,739 genes that do not change (FC 0.5–2) in total transcript abundance upon wounding (WT_Wound/WT_Unwound), 381 were more (FC ≥ 2) and 1,256 were less (FC ≤ 0.5) associated with the ribosomes (WT_Wound_TRAP/WT_Wound). **(B**,**C)** PANTHER GO Enrichment Analysis of the 1,636 transcripts that either increased **(B)** or decreased **(C)** in ribosomal association from **(A)**. Statistically significant GO terms were selected using Fisher exact test and Bonferroni correction for multiple testing (*P* < 0.05). Representative biological processes with fold enrichment above 1.5 are displayed. The full list of GO and genes in each category is provided in Supplementary Table 3.

As we begin to analyze the data, in order to simplify the analysis and to ensure that we are dealing with the genes that are differentially regulated exclusively at the level of ribosomal association and not at the level of transcription, transcripts that did not change in abundance after wounding were preselected from the WT total RNA-Seq data (Figure 4A). There were 19,739 genes that did not change (FC > 0.5 and < 2) in total transcript abundance upon wounding compared to unwounded controls (WT_Wound/WT_Unwound). These represent the majority (73.12%) of genes that were similar in transcript levels between the wound versus unwounded samples. Among these 19,739 transcripts, as many as 18,101 representing > 91% were not different when total mRNA (WT_Wound) and those associated with ribosomes (WT_Wound_TRAP) were compared, indicating that wounding did not have a colossal effect on mRNA association with ribosomes for a large swath of genes in the genome in 20 min after wounding in leaf tissue. However, still a significant number of genes (1,636) were either more (FC ≥ 2; 381 genes) or less (FC ≤ 0.5; 1,255 genes) preferentially associated with the ribosomes after wounding (WT_Wound_TRAP/WT_Wound) (Figure 4A). Within these 1,636 genes whose ribosomal versus total transcripts differ more than twofold in wounded leaves, a predominant number was changed toward less associated (1,255) than more associated (381) with the ribosomes, indicating that for a larger number of genes, translation activity could be reduced when cells are wounded despite no change in overall transcriptional activity (Figure 4A and Supplementary Table 2). Those transcripts fell into some distinctive functional groups according to GO terms assigned by the PANTHER classification system (see text footnote 5) (Figures 4B,C) (Gaudet et al., 2011). GO terms for those transcripts that were more associated with the ribosomes (FC ≥ 2; 381 genes) were as follows: “regulation of amino acid transport,” “mRNA transcription,” “regulation of ion transport,” “response to abiotic stimulus,” and “response to stress.” Those transcripts that were less associated with the ribosomes (FC ≤ 0.5; 1,256 genes) fell in the GO categories of “mitotic chromosome condensation,” “vesicle transport along actin-filament,” “microtubule-based movement,” “ATP synthesis coupled proton transport,” “cytokinesis by cell-plate formation,” “respiratory electron transport chain,” “oxidative phosphorylation,” and “photosynthesis” (Figure 3C). These GO term assignments are indicative of a shift in priority by the plant from those that are important for cell division and growth to transcription, stress response, and various transport function by wounding.

We also carried out similar GO classification studies with a set of genes that have not been preselected for transcriptional changes (Supplementary Figure 1). This was to have a more general view about the translatome regardless of their transcriptional inducibility by wounding. This was done both with unwounded (U) and wounded (W) WT samples. In total, 2,113 genes were found to be different in their abundance between ribosomal versus total mRNA fraction. Before wounding, there were 538 that were differentially regulated [FC (WT_U_TRAP/WT_U) ≥ 2 and ≤ 0.5, *p* < 0.05] that did not overlap with those from wounded samples. They were roughly evenly split between increased (286) or decreased (252) in their association with the ribosomes. Those more associated with ribosomes were involved in cell division and metabolism (copper ion, glutathione, sulfur, amino acid), whereas those less associated with ribosomes were involved in signal transduction (ARF, small GTPase, Ras) and DNA repair (Supplementary Figures 1B,E). In wounded leaves (WT_W_TRAP/WT_W), there was a similar number (617) of genes that were differentially expressed exclusively in the wounded tissues with more genes with transcripts that are less associated with ribosomes after wounding (385) than those more ribosome-associated (232) (Supplementary Figure 1A), consistent with what was observed earlier with the 19,739 preselected set (Figure 4A). GO terms for these less-ribosome–associating transcripts were also related to cell division, cell cycle, and DNA metabolic processes (Supplementary Figure 1F). Those transcripts more associated with ribosomes after wounding were related to various stress responses, cellular responses to oxygen levels, and transcription (Supplementary Figure 1C), similar to the results from the 19,739 preselect set (Figure 4A). The largest number of genes (958) that were differentially regulated between the ribosomal and total RNA fraction comparison fell under the category that maintained consistent ratio between the two fractions regardless of wounding state (the common area in the Venn diagram) (Supplementary Figure 1A). The predominant number (724 out of 958) in this group was less associated with the ribosomes, indicating that among those that are differentially partitioned between ribosome and total, more transcripts tend to be in the free state than engaged in translation. As will be described later, this phenomenon of a greater number of genes whose transcripts are in greater abundance in the total than in ribosomal fraction is exacerbated in the *b1b3* mutant.

### Direct Comparison of Translatomes Between WT Versus b1b3

**FIGURE 5:**
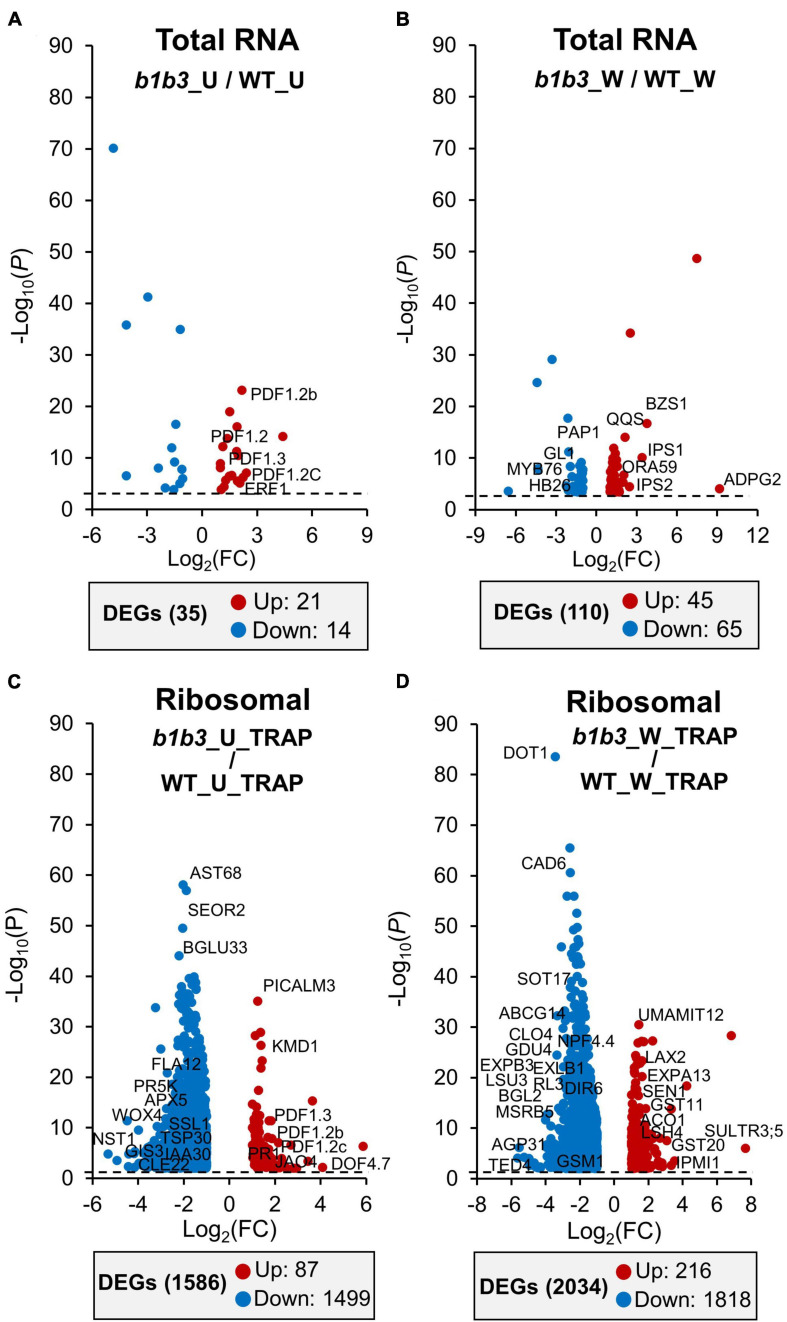
Transcript abundance comparisons between WT and *b1b3* in the total and TRAP RNA pools. **(A**,**B)** Volcano plots of differentially expressed genes (DEGs) in total RNA of unwounded (U) **(A)** and wounded (W) **(B)** samples of WT and *b1b3*. **(C**,**D)** DEGs in TRAP RNA preparations of U and W leaf samples. Comparisons of log_2_FC of FPKM ≤ −1 and ≥ 1 per indicated sample were plotted (*x*-axis). Dotted line indicates the *P* value cutoff of < 0.05. Names of representative genes are displayed. Full list of gene names is provided in Supplementary Table 4. The numbers of up-regulated and down-regulated DEGs are shown in the gray boxes below each graph.

Coming back to the main issue of the large influence the *b1b3* mutation has on both the wound phenotype and the proteome, the RNA-Seq and TRAP-Seq data for the two genotypes (WT and *b1b3*) were directly compared. A volcano plot of differentially expressed genes (DEGs) immediately illustrated the stark difference between the two genotypes (Figure 5 and Supplementary Table 4). First, when comparing only the total mRNA fractions of unwounded and wounded samples (*b1b3*_U/WT_U and *b1b3*_W/WT_W), there were only 35 and 110 DEGs, respectively, between the two genotypes (Figures 5A,B). This is a remarkably small number of DEGs given the large number of genes in the genome (∼30,000) and the relatively strong phenotypes displayed by *b1b3* plants upon wounding, although this had been predicted based on the earlier targeted gene expression studies showing that the marker gene transcript abundances did not differ much between the two genotypes (Poudel et al., 2016). Even the 145 (35 + 110) DEGs displayed a similar trend of changes; that is, those induced in one genotype were also induced in the other genotype (with only the degree of differences qualifying them as DEGs), and the same with the down-regulated genes, instead of displaying opposite trends. However, a completely different picture emerged when the TRAP-Seq datasets were compared between the two genotypes (*b1b3*_U_TRAP/WT_U_TRAP and *b1b3*_W_TRAP/WT_W_TRAP) (Figures 5C,D). The number of DEGs in unwounded and wounded datasets was 1,586 and 2,034, respectively. Most importantly, the changes that occurred were predominantly toward down-regulation in the *b1b3* (blue dots in Figures 5C,D). In the *b1b3*_U_TRAP/WT_U_TRAP comparison, there were 1,499 out of 1,586 DEGs that were less abundant in *b1b3* ribosome-associated transcripts, representing 94.5% of all DEGs in that comparison. Similarly, in the *b1b3*_W_TRAP v WT_W_TRAP comparison, 1,818 out of 2,034 DEGs (89%) were less abundant in the *b1b3*. This supports the hypothesis that the large reduction in proteome observed in *b1b3* (Figures 1, 2) may be in part caused by the reduction in number of transcripts associated with the ribosomes in *b1b3* compared to WT.

### Differential Focus on Cellular Function Between WT Versus b1b3 Translatome

Next, we looked at what kind of genes are in those DEGs between WT and *b1b3* translatome. DEGs in the unwounded (*b1b3*_U_TRAP/WT_U_TRAP) and wounded (*b1b3*_W_TRAP/WT_W_TRAP) TRAP-Seq comparisons were subject to GO Enrichment Analysis using PANTHER (Supplementary Figure 2 and Supplementary Table 6). Among the most enriched functional categories in the unwounded TRAP-Seq comparisons (*b1b3*_U_TRAP/WT_U_TRAP) were those related to vascular and secondary cell wall biogenesis (Supplementary Figure 2A). This shows that although unstressed *b1b3* does not display obvious growth or developmental defects (Poudel et al., 2016), there are basal differences in ribosome-associated transcripts that could potentially prime the plant for response to external stimuli (i.e., wounding). Glucosinolate metabolic genes were also highly enriched as down-regulated in this unwounded TRAP-Seq comparison (*b1b3*_U_TRAP/WT_U_TRAP).

**FIGURE 6:**
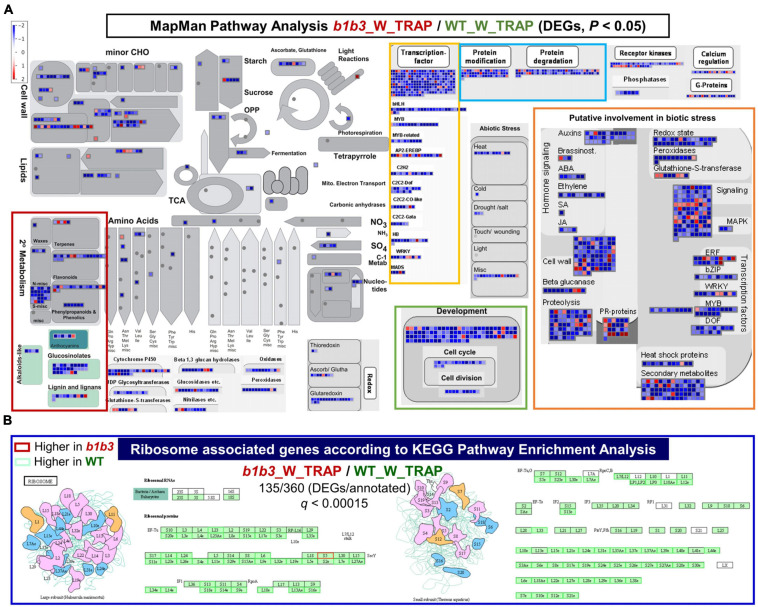
Pathway analysis of differentially expressed TRAP transcripts in wounded WT and *b1b3*. **(A)** MapMan pathways analysis of the *b1b3*_W_TRAP/WT_W_TRAP comparisons. All transcripts included in the figure met the *P* value cutoff (*P* < 0.05) and the FC cutoff (log_2_FC ≤ −1 and ≥ 1). Each colored square is representative of a single gene, and the color indicates either up- or down-regulation with scales from red to blue as shown in the key. **(B)** Ribosome-associated genes found to be most significantly (*q* < 0.00015) overrepresented in the *b1b3*_W_TRAP/WT_W_TRAP comparisons according to the KEGG Pathway Enrichment Analysis. Red and green boxes indicate higher and lower, respectively, expression in *b1b3* compared to WT. Cartoons represent large and small ribosomal protein complexes.

The trend of GO enrichment in vascular development, secondary cell wall biogenesis, cell cycle, and glucosinolate metabolism–related functional categories continued with the wounded WT and *b1b3* TRAP-Seq comparisons (*b1b3*_W_TRAP/WT_W_TRAP) (Supplementary Figure 2B). These wounded sample comparison data were further analyzed by MapMan (Thimm et al., 2004) and KEGG pathway analyses (Figure 6). The 2,034 genes identified earlier (Figure 5D) to be differentially expressed from the direct comparison of *b1b3*_W_TRAP/WT_W_TRAP including both up- and down-regulated in *b1b3* were mapped into various cellular pathways, including those relating to transcription factors, protein modification, protein degradation, development, cell cycle and division, primary and secondary metabolism, and biotic and abiotic stresses (Figure 6A). One striking aspect made visible from this analysis was that the less-ribosomal-association trend dominated across all listed pathways indicating that there was a large underrepresentation of transcripts associated with the ribosomes in *b1b3*. Among the transcription factors representing various classes were MYC4 and MYC5 that work redundantly with MYC2 and MYC3 to mediate multiple JA responses including root growth inhibition, specialized metabolite biosynthesis, and defense against insects (Chini et al., 2007; Kazan and Manners, 2008; Fernandez-Calvo et al., 2011; Figueroa and Browse, 2012; Song et al., 2017). Down-regulation of these functional pathways correlates well with the biochemical and growth phenotypes of wounded *b1b3*, namely, resistance to growth inhibition, reduced glucosinolates and other secondary metabolites, increased susceptibility to insects (Poudel et al., 2016), and reduced protein levels (Figures 1, 2).

One interesting class of gene transcripts identified by KEGG enrichment analysis that stood out to be differentially affected in their association with the ribosomes by the *b1b3* mutation was that related to ribosomes themselves (Figure 6B). Ribosomes are a complex assembly of RNA and RPs with at least ∼80 RP members making up a ribosome, and each RP unit is encoded by several paralogs in *Arabidopsis*. In wounded *b1b3*, a large fraction of the transcripts encoding RPs (135/360) are less associated with ribosomes. This may result in a significant reduction in certain ribosome combinations that may be involved in translating proteins of certain pathways in line with the increasing evidence of specialization of ribosomal function (Martinez-Seidel et al., 2020).

### Comparisons Between Transcriptome (RNA-Seq), Proteome, and Translatome (TRAP-Seq)

Finally, correlation between the three types of omics data was assessed using two approaches, correlation coefficient measurements and hierarchical clustering, both by using Morpheus tools (Supplementary Figure 3). Instead of using all data sets, data corresponding to the 1,956 genes that were identified by the proteomics approach (Figure 2), which was limiting among the three data sets, were used for this comparison. In other words, data were available for all three analyses (RNA-Seq, proteomics, and TRAP-Seq) for these 1,956 genes. To normalize across different types of data, log_2_(FC) values were used.

Data were grouped largely according to the types of analysis rather than by genotypes or treatments (Supplementary Figure 3). The transcriptomics data, regardless of genotype, gave correlation coefficient values (*r*) > 0.8, indicating a high level of correlation within the group. The correlations between the W_TRAP/U_TRAP of the TRAP-Seq datasets and the RNA-Seq datasets were relatively high at *r* > 0.77 for both genotypes. This is likely because albeit being TRAP-Seq data, the TRAP versus TRAP comparisons are more reflective of the transcriptional changes than the translational changes, which is measured by translational efficiency (ribosome-associated transcripts/total transcripts or TRAP-Seq/RNA-Seq) (Merret et al., 2017; Xu et al., 2017). The proteomics data for WT and *b1b3*, although appearing to group together in the figure (Supplementary Figure 3), had no correlation with each other (*r* < 0.1). This was true using either all 1,956 protein entries identified from the proteomics analysis (Supplementary Figure 3A) or a subset of 239 selected proteins whose abundance changed up or down twofold or more by wounding (Supplementary Figure 3B). The proteomics data for these 239 subsets had negative *r* scores reflecting the earlier observation of vast differences between the two genotypes (Figure 2). Next, the *r* values for the translatome data (TRAP/total RNA) against proteome and transcriptome were assessed. Somewhat contrary to our expectation, no correlation was observed between the translatome versus proteome comparisons (*r* < 0.02) for both genotypes, whether wounded or not. Similarly, no correlations were observed between (*r* < 0.1) the translatome datasets and the transcriptome datasets.

A similar trend of clustering among data sets emerged with hierarchical clustering and heatmap visualization (Supplementary Figure 3C). The transcriptome and translatome data clustered by themselves, whereas the proteome data for WT and *b1b3* formed their own independent branches. The translatome data clustered between the same genotypes rather than across different treatments (i.e., wound or unwound), indicating that the genotype had greater influence on ribosomal association of mRNA than wounding.

## Discussion

Various gene expression studies have been undertaken to investigate the transcriptional reprogramming occurring in plants after wounding or treatment with JA (Reymond et al., 2000; Lorenzo et al., 2004; Devoto et al., 2005; Mandaokar et al., 2006; Memelink, 2009; Pauwels et al., 2009; Attaran et al., 2014; Ikeuchi et al., 2017; Zander et al., 2020). The JA signaling pathway is responsible for up to 80% of the wound-induced transcriptome changes (Reymond et al., 2004; Gfeller et al., 2011). The molecular mechanism of JA controlled transcriptional regulation through COI1-JAZ coreceptor and transcription factors (e.g., MYC2/3/4/5) was elucidated at the mechanistic and structural level (Xie et al., 1998; Chini et al., 2007; Dombrecht et al., 2007; Thines et al., 2007; Sheard et al., 2010; Zhang et al., 2015; Howe et al., 2018; Chico et al., 2020). In contrast, the direct involvement JA signaling has in processes outside transcriptional control is largely unknown. Although the data presented in this research also do not determine the molecular mechanism of how JA might be involved in posttranscriptional regulation of genetic information flow from DNA to protein, the data showing changes in proteome and ribosomal association of transcripts caused by genetic mutations that alter the JA profile in *b1b3* have offered a first peek at the influence of the JA pathway on these processes.

The striking phenotypes of the *b1b3* mutant could not be easily explained by its transcriptional differences compared to WT (Poudel et al., 2016), which is in itself surprising given how the major way in which JA exerts its effect on plant physiology is via transcription (Kazan and Manners, 2008; Howe et al., 2018). Our transcriptome data (Figure 5) again confirmed earlier qRT-PCR results of marker genes showing normal behavior in terms of transcriptional response in the mutant (Poudel et al., 2016). Exogenous application of JA also resulted in normal induction of marker gene expression in *b1b3*, discounting the possibility of its defect in molecular perception or transcriptional mechanism of JA (Poudel et al., 2016, 2019). The possibility of *b1b3* phenotypes being merely pleiotropic or random can be raised, but the mutant displaying phenotypes consistently in most, if not all, classical JA-dependent wound responses such as growth inhibition, anthocyanin accumulation, trichome biogenesis, specialized metabolite induction, and insect resistance, albeit in an exact opposite direction of the expectation, implied that the phenotypes must still be somehow influenced by the altered JA pathway in *b1b3*.

Protein changes provided an alternative mechanism to explain the gap between the normal transcription and abnormal downstream phenotypes of *b1b3* (Figure 1). The proteomics data confirmed that the unusual decrease in marker proteins in the wounded *b1b3* was not an isolated event restricted to a few JA marker proteins but a more widely spread phenomenon in the mutant (Figure 2). However, it is important to note that although there is a prevalent reduction in abundance of many proteins in *b1b3*, still the vast majority (>90%) of the proteome was unchanged from the WT at the given moment. This indicates a certain degree of specificity to the effect of the mutation on the proteome. The identity of those proteins repressed by wounding in *b1b3* (compared to WT) was also not restricted to typical JA-responsive marker genes/proteins, but rather was distributed across several functional classes. Somewhat related to this observation, earlier proteomic studies by Gfeller et al. (2011) in *Arabidopsis* have found that many proteins differentially regulated by wounding were not regulated by transcription (Gfeller et al., 2011). However, a majority of the wound-regulated proteins they found were deregulated by blocking JA biosynthesis. Collection of these differentially expressed proteins may be useful in the future to elucidate the protein-level regulation of JA during wound responses. Selection of proteins for such purpose will require additional work to confirm the results provided by our first-pass proteomic study. The degree of protein level changes must also be determined through more quantitative targeted and comprehensive proteomic studies with higher-stringency selection criteria.

One potential way protein levels could be altered is through translation. We found that there is a large change in the ribosomal-associated pool of mRNA after wounding in WT. Many transcripts (>90%) did not change their ribosomal association after wounding at 20 min, but among the 10% that did change (1,638), vast majority (1,256 or 77%) became less associated with the ribosomes. There was an increase in the ribosomal association of transcripts relating to mRNA transcription and abiotic stimulus, whereas there was a decrease in those relating to cell division and energy metabolism (Figure 4 and Supplementary Figure 1). A similar trend has been observed in other stress responses (Branco-Price et al., 2008; Merchante et al., 2017; Merret et al., 2017; Meteignier et al., 2017; Martinez-Seidel et al., 2020). This also shows that not only is there transcriptional adjustment in activating defense while pausing growth in response to wounding, but similar regulation is found in terms of ribosomal association.

As many as 1,586 and 2,034 gene transcripts in the unwounded and wounded samples, respectively, were differentially regulated in the ribosomal fraction in a direct comparison between WT and *b1b3* (Figure 5). While those numbers may be considered to be minor in a backdrop of 25,000 to 30,000 genes in the *Arabidopsis* genome, they were significant compared to the total transcript comparisons that yielded a mere 35 in unwounded and 110 in wounded DEGs between the two genotypes. More strikingly, the majority (1,499 and 1,818 representing 95% and 89%, respectively) of these DEGs in the ribosomal fraction had a smaller number of ribosome-associated transcripts occurring in *b1b3* compared to WT. This is in line with the reduced protein abundance seen in the *b1b3* proteome (Figure 2) and suggests an exacerbating effect of *b1b3* mutation on wound-induced reduction of ribosome-associated transcripts seen in WT (Figure 4). Most notable changes in the wounded *b1b3* ribosomal fraction were in the down-regulation of transcripts associated with specialized metabolism, development, protein degradation, and abiotic and biotic stress (Figure 6A). These pathways are related to various phenotypes exhibited by *b1b3* plants (Poudel et al., 2016).

The more difficult question to answer lies on the issue of specificity. As mentioned earlier, the numbers of differentially expressed ribosome-associated transcripts between wounded WT and *b1b3* were in the order of few thousands (Figure 5D). These are not evenly distributed across all functional categories, which would have indicated random reduction in overall ribosome association in *b1b3* but are rather enriched in the aforementioned functional classes indicating specificity. Generally speaking, at least two types of translational regulation can be considered—global regulation and gene−specific control—although they are not always inseparable (Merchante et al., 2017). During global or whole-genome translation regulation, which can happen during stress conditions, such as hypoxia, there are several mechanisms at work including the phosphorylation of poly(A)−binding proteins and elongation initiation factors (Browning and Bailey-Serres, 2015). Global translation regulation can also be achieved through exchanging protein composition of the general translational machinery (Merchante et al., 2017; Martinez-Seidel et al., 2020) (which will be discussed in more detail below). Gene-specific translational regulation refers to uneven translational activity among specific mRNAs that create disproportional representation of proteins encoded by those transcripts compared to others in the cell. Some known mechanisms of this type of regulation involve small RNAs, specific RNA-binding proteins and translation factors, small molecules, and *cis*-regulatory elements internal and external of the mRNA (Brodersen et al., 2008; Liu et al., 2013; Cui et al., 2015; Merchante et al., 2015, 2017; Hou et al., 2016). Although yet to be completed, our ongoing investigation into the conserved sequence features of mRNA repressed in the ribosome-associated fraction of *b1b3* mRNA could potentially add to the mechanisms conferring the specificity.

Protein composition of ribosomal super-complex can also play a role in specific translational regulation (Martinez-Seidel et al., 2020). As many as 135 genes encoding RPs were differentially expressed in wounded *b1b3*, predominantly less abundant in the mutant than WT (Figure 6B). The proteomics data could not independently verify many of the TRAP-seq results probably due to multiple factors, including differences between steady-state level of proteins and more transient ribosome-associated mRNAs levels. Differences in tissue sampling times, different data sizes, and similarity among RP paralogs could have also contributed to the proteomic detection failure. However, evidence supporting functional heterogeneity of RPs as opposed to homogenous mRNA translational machineries have increased over the years in plants (Martinez-Seidel et al., 2020), similar to cases in yeast and mammals (Gilbert, 2011; Shi et al., 2017). There can be two to seven paralogs per RP for a total of more than 230 proteins identified as RPs in the *Arabidopsis* genome rendering an immense number (10^34^) of possible ribosomal combinations (Barakat et al., 2001; Browning and Bailey-Serres, 2015; Martinez-Seidel et al., 2020). Although the basic functions of ribosomes are likely conserved, it is not unreasonable to expect that this ribosomal heterogeneity would contribute to functional and regulatory specialization. In fact, there is growing evidence supporting differential regulation of a subset of RPs at the transcriptional, posttranscriptional, translational, and posttranslational levels in response to developmental cues, environmental conditions, and phytohormones (Barakat et al., 2001; McIntosh and Bonham-Smith, 2006; Muench et al., 2012; Asensi-Fabado et al., 2017; Schepetilnikov and Ryabova, 2017; Wang et al., 2017; Cheong et al., 2020).

Other modes of regulations for translation that could confer specificity include a set of proteins called ribosome-inactivating proteins (RIPs) that irreversibly inhibit protein translation by depurination of ribosomal RNA (Barbieri et al., 1993; Chaudhry et al., 1994; Reinbothe et al., 1994; Nielsen and Boston, 2001; Jiang et al., 2008; Bolognesi et al., 2016). The expression of several RIPs is regulated by JA and abiotic and biotic stress, thereby alluding to their potential regulatory roles as a regulator of various environmental cues and hormone signaling (Jiang et al., 2008; Rustgi et al., 2014; Genuth and Barna, 2018; Zhu et al., 2018). Various types of RIPs have been reported (Stirpe and Battelli, 2006; Bolognesi et al., 2016), the most prominent example being ricin from *Ricinus communis* L. (caster bean), but none have been reported in *Arabidopsis* and hence have not been detected among our data. However, their wide distribution among plant species would predict existence of their functional counterpart in *Arabidopsis*.

As such, there are a number of potential mechanisms to explain how a specific pool of translating mRNAs can be differentially regulated, but how that exactly relates to the *b1b3* mutation is still not known. The immediate metabolic defect caused by the mutation is changes in JA-Ile and 12OH-JA-Ile hormone levels. The molecular target of 12OH-JA-Ile was shown to be the COI1-JAZ coreceptor system. This was shown by 12OH-JA-Ile’s ability to promote molecular interaction between COI1 and JAZ *in vitro* and *in silico*, and that its signaling effect *in planta* can be blocked by mutation in COI1 (Koo et al., 2011; Jimenez-Aleman et al., 2019; Poudel et al., 2019). However, this signaling system that mainly regulates transcription is not altered in *b1b3*. This leads to at least several hypotheses. One, there are alternative molecular targets of these JAs or their derivatives related to ribosomal loading or translational control. Alternatively, there could be alternative novel substrates for CYP94Bs besides JA metabolites. In this case, those alternative substrates should have as profound effect on the wound response as JAs in order to explain the broad spectrum of JA-related *b1b3* phenotypes. In addition, small transcriptional changes in the *b1b3* mutant could still lead to downstream production (or inhibition) of effectors that could target translation. The general lack of a mechanistic understanding of many of the translational mechanisms in plants is both a challenge and an opportunity to understand plant adaptation to their environment. The *b1b3* mutant can be used as a tool to further study translational regulation and its interplay with stressors such as wounding.

There have been mixed reports about the correlation between transcriptomics and proteomics data, but the dominant consensus has been that they are generally not correlated very well (Baerenfaller et al., 2008; Gfeller et al., 2011; Fernie and Stitt, 2012; Walley et al., 2016; Fan et al., 2017; Zander et al., 2020). Our analysis also showed poor correlation between transcriptome and proteome data. This may have been in part caused by different time points used for those two analyses. However, even comparison between current proteomics and transcriptome data generated at various timepoints produced in-house still resulted in low correlation. This observation advises caution against common practices among researchers to explain phenotypic data based solely on gene transcription and underscores the importance of taking into account the discordant behavior between transcript and protein abundance. Regarding the translatome data, our intuitive expectation was that they would capture somewhat intermediary dynamics between the transcriptome and proteome. However, the translatome data formed their own cluster and did not show clear correlation with neither the transcriptome nor proteome data. However, this may again be due to different sampling times. Our conclusion and discussion, in general, are based on observations made at particular time points in a particular tissue type (i.e., leaf). Higher time resolution data at various developmental stages in different tissue types as well as information about their kinetic behavior are needed to provide a more accurate picture of the collective changes occurring after wounding stress in the future.

## Data Availability Statement

The datasets presented in this study can be found in online repositories. The names of the repository/repositories and accession number(s) can be found below: Bioproject SRA data: PRJNA692417.

## Author Contributions

AKi and AKo designed experiments. AKi performed experiments. RH performed computational data analysis. AKi, RH, and AKo analyzed the data and wrote the manuscript. All authors contributed to the article and approved the submitted version.

## Conflict of Interest

The authors declare that the research was conducted in the absence of any commercial or financial relationships that could be construed as a potential conflict of interest.

## Footnotes

1 http://www.biovenn.nl/index.php

2 https://software.broadinstitute.org/morpheus/

3 https://mapman.gabipd.org/

4 https://www.genome.jp/kegg/

5 http://pantherdb.org

## Supplementary Material

Supplementary Figure 1 GO term classification of genes with transcripts that are differentially associated with the ribosomes (TRAP/total RNA) in unwounded and wounded Wt leaves.

Supplementary Figure 2 GO term classification of genes that are less-associated with ribosomes in *b1b3* through a direct comparison with unwounded and wounded Wt TRAP datasets.

Supplementary Figure 3 Correlation coefficient chart and hierarchical clustering map illustrating the relationship between the transcriptome, translatome, and proteome of Wt and *b1b3*.

Supplementary Table 1 Proteins changed by wounding in Wt and *b1b3* leaves.

Supplementary Table 2 Genes with transcripts exclusively changing their ribosomal association after wounding without changing their net transcription.

Supplementary Table 3 GO Enrichment Analysis of genes with transcripts that do not change transcriptionally but are differentially associating with the ribosomes (TRAP/total RNA) in unwounded and wounded Wt leaves.

Supplementary Table 4 Transcript abundance difference between Wt and *b1b3* in the total and TRAP RNA pools.

Supplementary Table 5 GO Enrichment Analysis of genes with transcripts that are differentially associating with the ribosomes (TRAP/total RNA) in unwounded and wounded Wt leaves.

Supplementary Table 6 GO Enrichment Analysis of genes that are less-associated with ribosomes in *b1b3* through a direct comparison with unwounded and wounded Wt TRAP datasets.
