# Supplementary material for: Differential Regulation of the Ribosomal Association of mRNA Transcripts in an Arabidopsis Mutant Defective in Jasmonate-Dependent Wound Response
Source: Front Plant Sci. 2021 Mar 11;12:637959. doi: 10.3389/fpls.2021.637959 (PMC7990880; doi:10.3389/fpls.2021.637959)
Supplement: Supplementary Figure 1 — GO term classification of genes with transcripts that are differentially associated with the ribosomes (TRAP/total RNA) in unwounded and wounded Wt leaves. [file Data_Sheet_1.PDF]

Differential regulation of the ribosomal association of mRNA transcripts in an Arabidopsis mutant defective in jasmonate-dependent wound response

Athen Kimberlin[1,2†], Rebekah E. Holtsclaw[1,2†], and Abraham J. Koo[1,2*]

[1]Department of Biochemistry, University of Missouri, Columbia, MO 65211, USA

[2]Interdisciplinary Plant Group, University of Missouri, Columbia, MO 65211, USA

Supplementary Figure S1. GO term classification of genes with transcripts that are differentially associating with the ribosomes (TRAP / total RNA) in unwounded and wounded WT leaves.

Supplementary Figure S2. GO term classification of genes that are less-associated with ribosomes in *b1b3* through a direct comparison with unwounded and wounded WT TRAP datasets.

Supplementary Figure S3. Correlation coefficient chart and hierarchical clustering map illustrating the relationship between the transcriptome, translatome, and proteome of WT and *b1b3*.

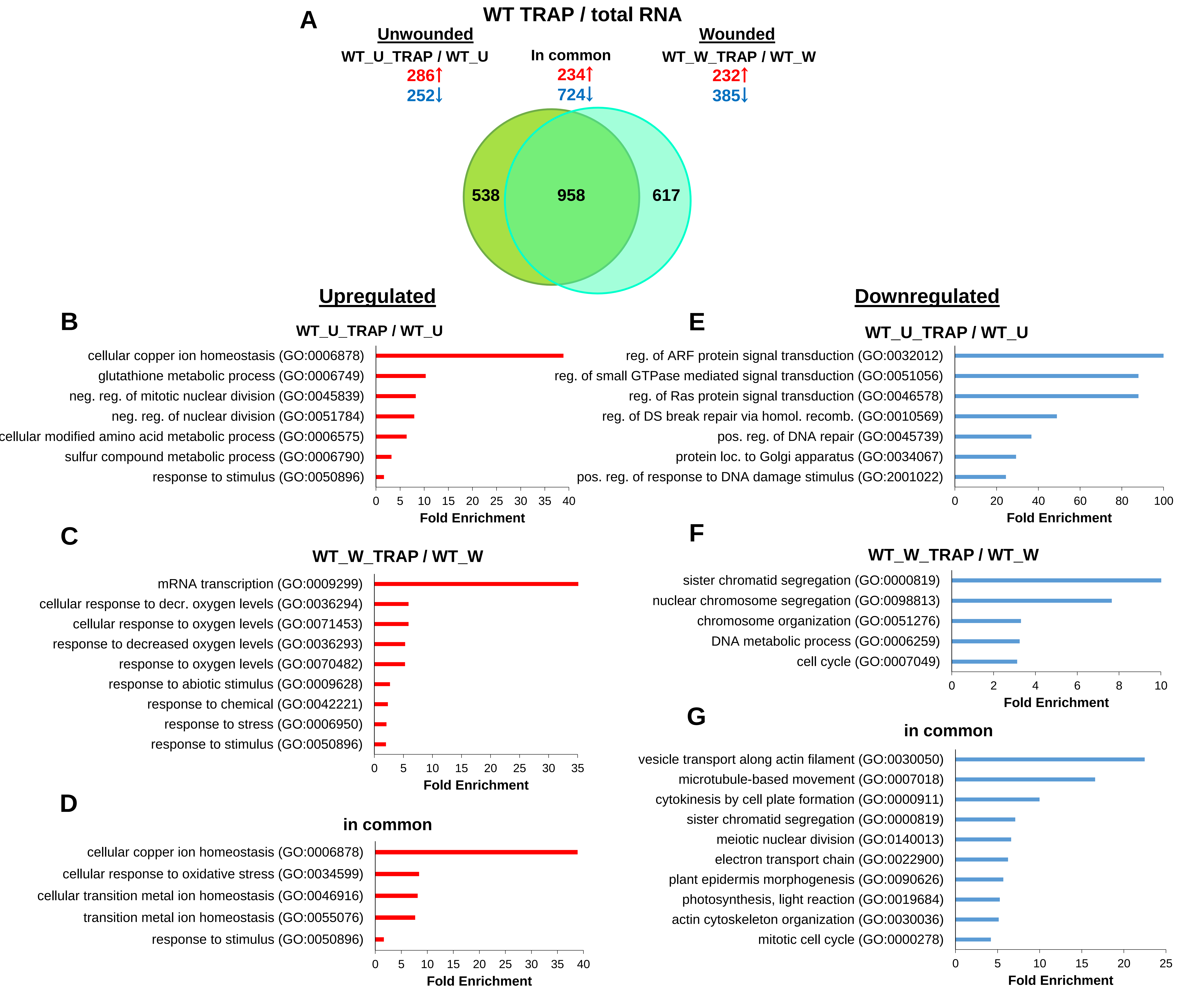

Supplementary Figure S1. GO term classification of genes with transcripts that are differentially associating with the ribosomes (TRAP / total RNA) in unwounded and wounded WT leaves. **(A)** Venn diagram summary of differentially expressed transcripts ($log_2FC > 1$ (up) or < -1 (down)) in unwounded (WT_U_TRAP / WT_U) or wounded (WT_W_TRAP / WT_W) samples. **(B-G)** GO terms and fold enrichment for those transcripts upregulated **(B-D)** or downregulated **(E-G)** from the unwounded (WT_U_TRAP / WT_U) **(B,E)** or wounded (WT_W_TRAP / WT_W) **(C,F)** sample comparisons. Those that are in commonly up or down regulated before and after wounding are shown separately **(D,G)**. The GO enrichment analysis was done by Panther using fisher's exact test and Bonferroni correction for multiple testing ($P < 0.05$). Representative biological processes with fold enrichment > 1.5 are displayed. The full list of GO and genes in each category is provided in Supplementary Table S5.

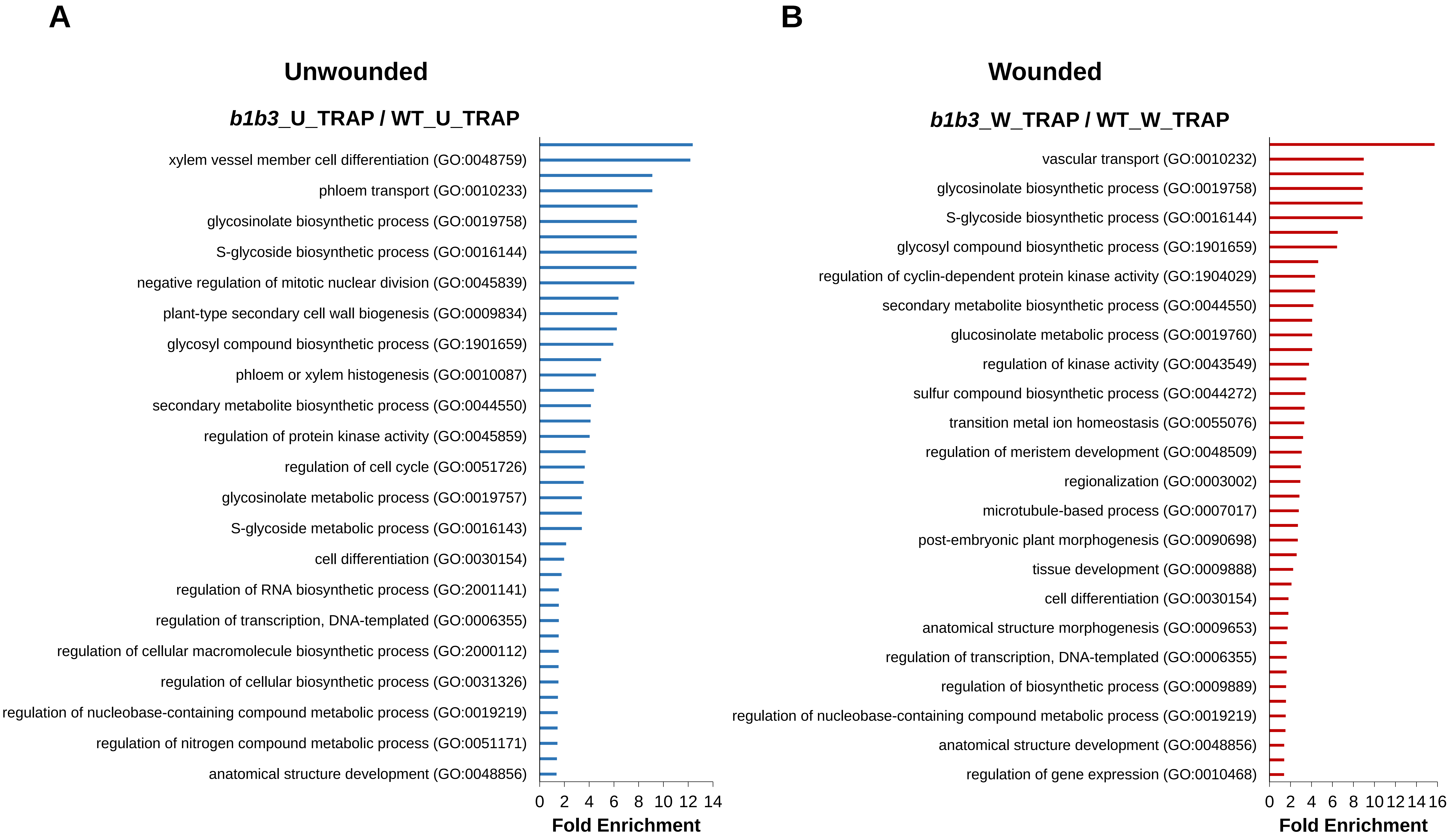

Supplementary Figure S2. GO term classification of genes that are less-associated with ribosomes in *b1b3* through a direct comparison with unwounded and wounded WT TRAP datasets. **(A-B)** GO terms and fold enrichment for those transcripts less abundant in b1b3 in the unwounded *b1b3*_U_TRAP / WT_U_TRAP **(A)** or wounded b1b3_W_TRAP / WT_W_TRAP **(B)** comparisons, respectively. The GO enrichment analysis was by Panther using fisher's exact test and Bonferroni correction for multiple testing (P < 0.05). Representative biological processes with fold enrichment > 1.5 are displayed. The full list of GO and genes in each category is provided in Supplementary Table S6.

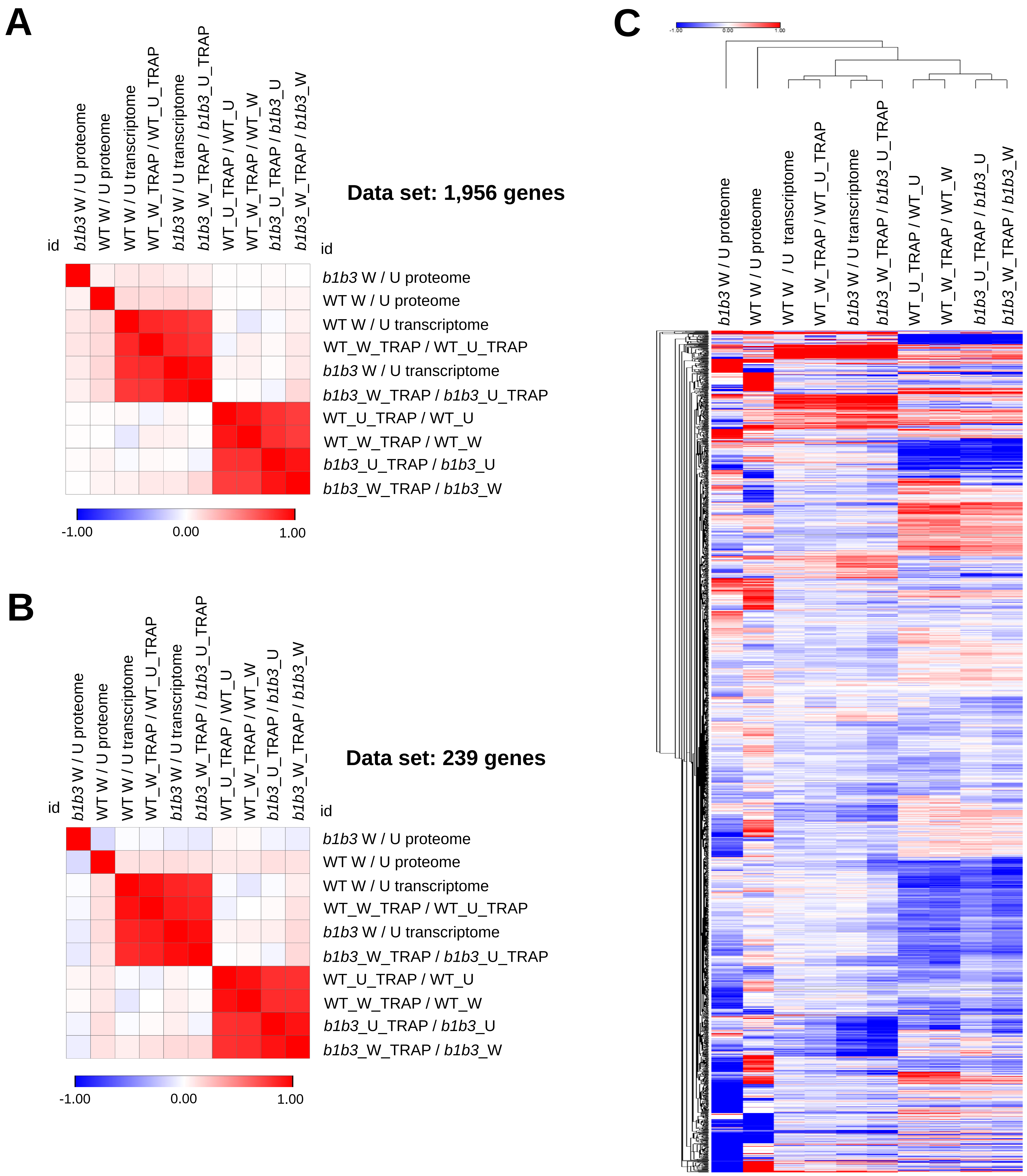

Supplementary Figure S3. Correlation coefficient chart and hierarchical clustering map illustrating the relationship between the transcriptome, translatome, and proteome of WT and *b1b3*. **(A,B)** Correlation coefficient was calculated by Morpheus tool that calculates the linear association between sets of values. An *r* value of 1 perfect correlation (red), and an *r* value -1 (blue) indicates a perfect anti-correlation. 1,956 protein-encoding genes identified **(A)** or 239 of those that changed up or down 2-fold by wounding **(B)** from the proteomics experiment were used as data to compare across corresponding data sets from RNA-Seq and TRAP-Seq. **(C)** Hierarchical clustering was done using the online Morpheus tool with Euclidean distance with complete linkage setting. The dendrograms illustrate relationship between the omics datasets (top) and the genes being clustered.
